# Defect Structure and Oxide Ion Conduction of Potassium Ion Substituted CaWO_4_

**DOI:** 10.3390/ma11071092

**Published:** 2018-06-27

**Authors:** Shigeomi Takai, Shinya Shitaune, Toshifumi Sano, Hitoshi Kawaji, Takeshi Yabutsuka, Takao Esaka, Takeshi Yao

**Affiliations:** 1 Graduate School of Energy Science, Kyoto University, Yoshida-Honmachi, Sakyo-ku, Kyoto 606-8501, Japan; sano.toshifumi.85w@st.kyoto-u.ac.jp (T.S.); yabutsuka@energy.kyoto-u.ac.jp (T.Y.); 2 Graduate School of Engineering, Tottori University, Koyama-cho Minami, Tottori 680-8552, Japan; unya.a.a.keropon@outlook.jp (S.S.); tko_esaka@chem.tottori-u.ac.jp (T.E.); 3 Laboratory for Materials and Structures, Tokyo Institute of Technology, Nagatsuta-Cho, Midori-ku, Yokohama 226-8503, Japan; kawaji@msl.titech.ac.jp; 4 Institute of Advanced Energy, Kyoto University, Gokasho, Uji, Kyoto 611-0011, Japan; t_yao@hera.eonet.ne.jp; 5 National Institute of Technology, Kagawa College, Chokushi-cho, Takamatsu, Kagawa 761-8058, Japan

**Keywords:** CaWO_4_, oxide ion conductor, oxide ion vacancy, high-temperature XRD

## Abstract

We have prepared Ca_1−x_K_x_WO_4−x/2_ solid solutions with the Scheelite-type structure to investigate high-temperature electrochemical properties. Room-temperature X-ray diffraction suggested the solid solution range was *x* ≤ 0.2, since the second phase presumably of K_2_WO_4_ was detected for *x* = 0.3. For all the substituted samples up to *x* = 0.4, a large jump in conductivity has been observed around 500 °C. At higher temperatures, oxide ion conduction is found to be predominant even for *x* = 0.4, exceeding the solution limit estimated from the room-temperature XRD. The conductivity at high temperature is essentially proportional to the amount of substituted potassium ions up to *x* = 0.4, indicating that oxide ion conduction is associated with the formed oxide ion vacancy. High-temperature X-ray diffraction detected no apparent change in lattice parameters around 500 °C for *x* = 0.1, and the remaining second phase seems to be incorporated into the Scheelite lattice at high temperatures.

## 1. Introduction

Considerable attention has been focused upon the oxide ion conductors for the application of the electrolyte of SOFC (Solid Oxide Fuel Cell) [1,2,3]. Since the major electrolyte materials of SOFC, such as stabilized zirconia or doped ceria, belong to the Fluorite-type structure; Scheelite-type structured materials derived from the Fluorite-type are a potential candidate for the oxide ion conductors [4]. We have intensively studied the PbWO_4_-based oxide ion conductors with the Scheelite-type structure [5,6,7,8]. When lanthanum ions are partly substituted into the lead site of PbWO_4_, forming oxide ion interstitials such as Pb_1−x_La_x_WO_4+x/2_, high oxide ion conduction appears at elevated temperatures. Such a defect structure has been confirmed by the powder density measurements [6]. On the other hand, CaWO_4_ with the mineral name of “Scheelite” is unlikely to form a solid solution by lanthanum substitution to create oxide ion interstitials. Nevertheless, in recent years, we have found that Cs-substituted CaWO_4_ with the form Ca_1−x_Cs_x_WO_4−x/2_ exhibits oxide ion conduction at elevated temperatures [9]. Since most oxide ion conductors with the Fluorite-type employ oxide ion vacancy for the oxide ion diffusion, mono-valent ion substitution for CaWO_4_ would be a promising strategy for exploring the new type of oxide ion conductors. Considering that the ionic radius of the K^+^ ion (165 pm for 8-coordinate) is closer to that of Ca^2+^ (126 pm) rather than Cs^+^ (188 pm) [10], potassium substitution is more appropriate for the solid solution formation with CaWO_4_. In the present study, we prepared the solid solution of Ca_1−x_K_x_WO_4−x/2_ to investigate the high-temperature electrochemical properties, and thereafter, high-temperature X-ray diffraction experiments were carried out emphasizing the phase transition phenomena.

## 2. Materials and Methods

Ca_1−x_K_x_WO_4−x/2_ samples were prepared by conventional solid-state reaction method, and were started using CaCO_3_, H_2_WO_4_, and K_2_CO_3_. The calcining and sintering temperatures were selected as 800 and 1000 °C, respectively, as per the previous Cs-substituted system [9]. The obtained samples were analyzed by X-ray diffraction with CuK_α_ radiation (XRD-6000; Shimadzu, Kyoto, Japan). Conductivity measurements were carried out by means of two-probe AC method, and oxygen gas concentration cells were constructed using the sample discs as electrolytes to evaluate the charge carrier. Pt electrodes were used for electrochemical measurements. In the present study, TG-DTA (DTG-50; Shimadzu, Kyoto, Japan) experiments up to 1000 °C have also been carried out to investigate the phase transition of the sample.

High-temperature X-ray diffraction experiments (AXS D8 Discover; Bruker, Billerica, MA, USA) up to 700 °C were then carried out for *x* = 0.1 and 0.4 to investigate the structural variation with temperature. The diffracted X-ray was collected by a 2D-detector in the diffractometer for 180 s per measurement. Lattice parameters were refined by the Rietveld method using RIETAN code [11] assuming the Scheelite-type structure with the space group of *I*4_1_/*a*.

## 3. Results and Discussion

**Figure 1:**
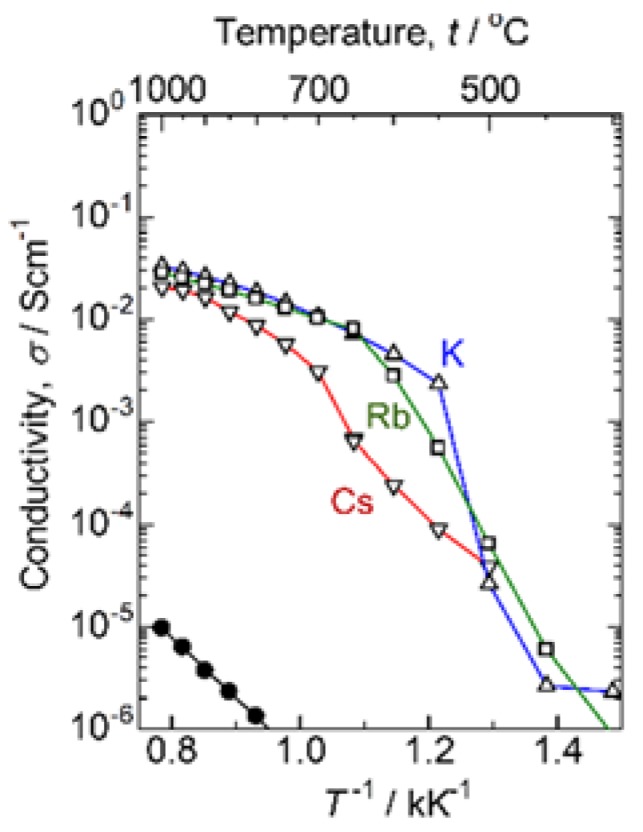
Arrhenius plots of conductivity for Ca_0.9_A_0.1_WO_3.9_. △: A = K, ☐: Rb, and ▽: Cs. Conductivity of the pristine CaWO_4_ is also plotted by closed circles (●).

We first carried out the preliminary experiments to check the solid solubility and electric conductivity for various alkaline ion substituted CaWO_4_s with the form Ca_1−x_A_x_WO_4−x/2_ (A: alkaline ion). From the room-temperature X-ray diffraction experiments, the solid solution limits were determined as *x* = 0.15, 0.10, 0.20 for Cs-, Rb-, and K-substitutions, respectively. As for Na-substitution, the solid solution cannot be obtained even for *x* = 0.05. We thus compared the high-temperature electric conductivities in these three compounds substituted by Cs^+^, Rb^+^ and K^+^ ions fixing the concentration as *x* = 0.1. Figure 1 compares the Arrhenius plots of conductivity for various alkaline ion substituted samples of Ca_1−x_A_x_WO_4−x/2_ (*x* = 0.1). While the Cs-substituted system gradually decreases the conductivity with reducing temperatures, the Rb- or K-substituted systems keep the relatively higher conductivity down to the intermediate temperatures with a drop in conductivity around 600 or 500 °C. On the other hand, all the substituted systems possess the similar conductivities as well as the similar slopes above 900 °C. Namely, keeping the higher conductivity down to the intermediate temperature exhibits for the K-substituted system. Accordingly, we carried out the electrochemical and structural studies focusing on the K-substituted CaWO_4_.

**Figure 2:**
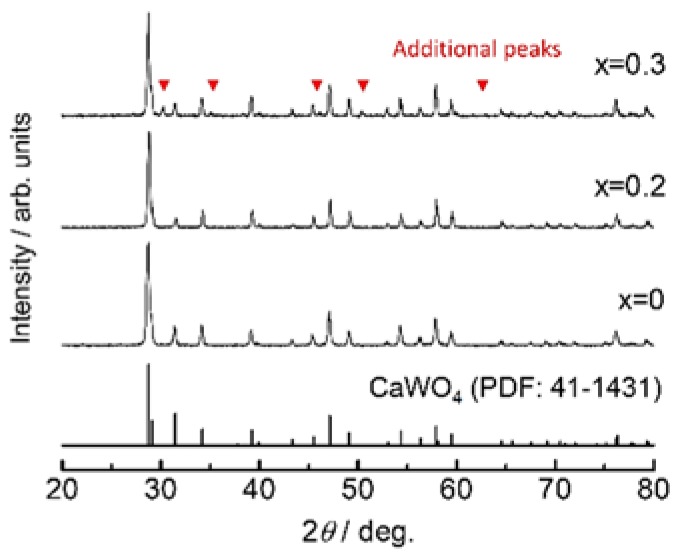
X-ray diffraction patterns of Ca_1−x_K_x_WO_4−x/2_ obtained at room temperature.

**Figure 3:**
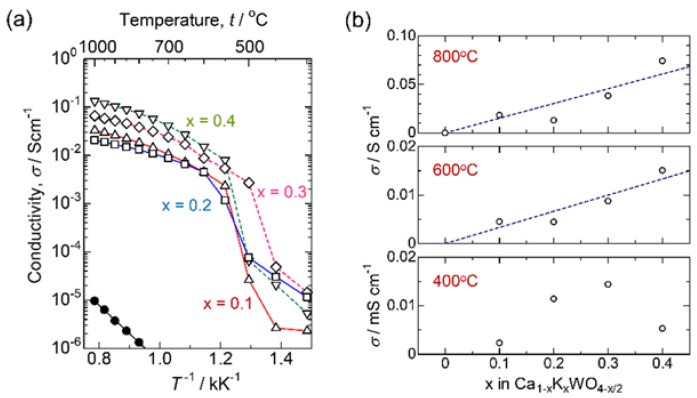
(**a**) Arrhenius plot of conductivity for Ca_1−x_K_x_WO_4−x/2_. ●: *x* = 0, △: 0.1, ☐: 0.2, ¯: 0.3, and ▽: 0.4; (**b**) Conductivity isotherms of Ca_1−x_K_x_WO_4−x/2_ at 400, 600, and 800 °C.

**Figure 4:**
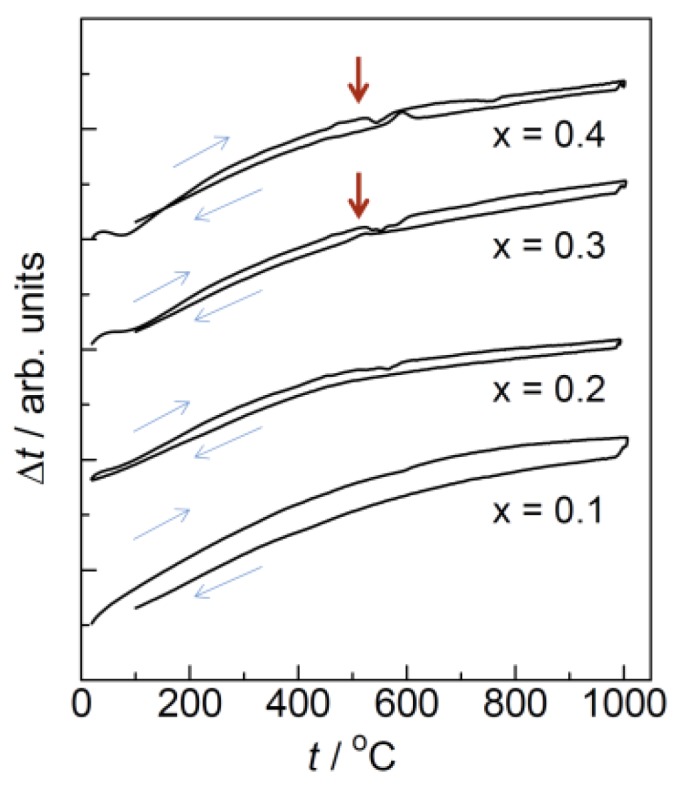
DTA traces of Ca_1−x_K_x_WO_4−x/2_. Red arrows indicate the small endothermic peak observed in the heating direction.

Figure 2 shows the X-ray diffraction patterns of Ca_1−x_K_x_WO_4−x/2_ measured at room temperature. While all the diffraction peaks can be assigned as the Scheelite-type structure for *x* ≤ 0.2, some additional peaks presumably due to K_2_WO_4_ (PDF: 21-1010) or K_4_WO_5_ (PDF: 24-0904) are observed at *x* = 0.3. Therefore, the solid solution range is evaluated as *x* ≤ 0.2 for Ca_1−x_K_x_WO_4−x/2_. The Arrhenius plots in conductivity measured for Ca_1−x_K_x_WO_4−x/2_ (*x* = 0–0.4) are represented in Figure 3a. As plotted for *x* = 0.1 in Figure 1, all the substituted samples exhibit the conductivity enhancement from CaWO_4_ by more than three orders of magnitude. Moreover, a large jump in conductivity is also observed around 500 °C. It should be noted that, at the higher temperature region, conductivity enhancement continues even after exceeding the solubility limit of *x* = 0.2. We assumed that excess potassium ions are incorporated into the Scheelite lattice around 500 °C to form oxide ion vacancy as the nominal form of Ca_1−x_K_x_WO_4−x/2_ showing a conductivity jump. DTA measurements were then carried out to investigate this phenomenon. The obtained DTA traces are represented in Figure 4. For *x* = 0.3 or 0.4, a small endothermic peak is observed around 500 °C in the heating direction, suggesting that formation of the solid solution would occur at this temperature. Moreover, even for *x* = 0.2, a very small peak is still detected at this temperature.

**Figure 5:**
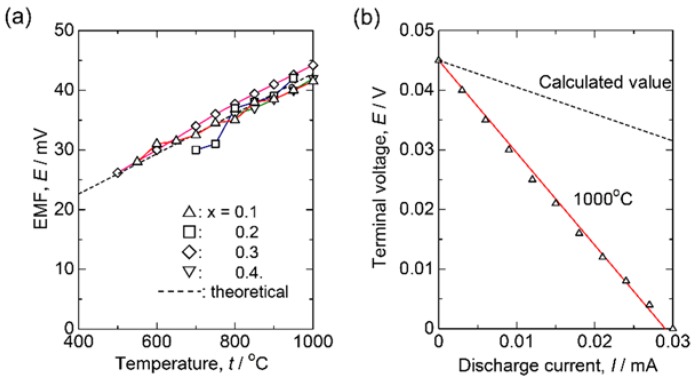
(**a**) EMFs of the oxygen gas concentration cells as follows using Ca_1−x_K_x_WO_4−x/2_ sample discs as the electrolyte. Air (*P*(O_2_) = 0.21 atm) | Ca_1−x_K_x_WO_4−x/2_ | O_2_ gas (*P*(O_2_) = 0.21 atm). △: *x* = 0.1, ☐: 0.2, ¯: 0.3, and ▽: 0.4. Dashed line denotes the Nernstian value; (**b**) Discharge plot of the oxygen gas concentration cell employing Ca_1−x_K_x_WO_4−x/2_ (*x* = 0.1) measured at 1000 °C. The calculated line was assuming only the ohmic loss.

To determine the charge carrier in this system, oxygen gas concentration cells are constructed using the sample as an electrolyte. The anode and cathode gasses selected were air (*P*(O_2_) = 0.21 atm) and pure O_2_. The measured EMFs (Electromotive Forces) are plotted in Figure 5a, where the calculated EMF, assuming Nernst equation, is:

$$\mathrm{EMF}=\frac{R\cdot T}{4F}\ln\frac{P_{O_{2}}\left(1\;\mathrm{atm}\right)}{P_{O_{2}}\prime \left(0.21\;\mathrm{atm}\right)}$$

and is indicated by the dashed line. Since the measured EMFs agree well with the calculated one, the predominant charge carrier is not electron but ion. In addition, since the constant current can be drawn from the cell showing linear decrease in terminal voltage with the current as Figure 5b, the charge carrier should be the oxide ions. In Figure 5a, the more interesting aspect is that EMFs obtained for the *x* = 0.3 or 0.4 beyond the solution limit also coincide with the Nernstian value. To clarify the compositional dependence of the conductivity, the measured electric conductivity is plotted versus potassium concentration as in Figure 3b. At 400 °C, conductivity increases with the potassium composition up to *x* = 0.3, followed by a drop at *x* = 0.4. However, the linear relationship between conductivity and potassium concentration can be seen at 600 or 800 °C. Therefore, excess potassium ions are supposed to be incorporated into the Scheelite lattice to form oxide ion vacancy around 500 °C, which allows the high oxide ion diffusion.

**Figure 6:**
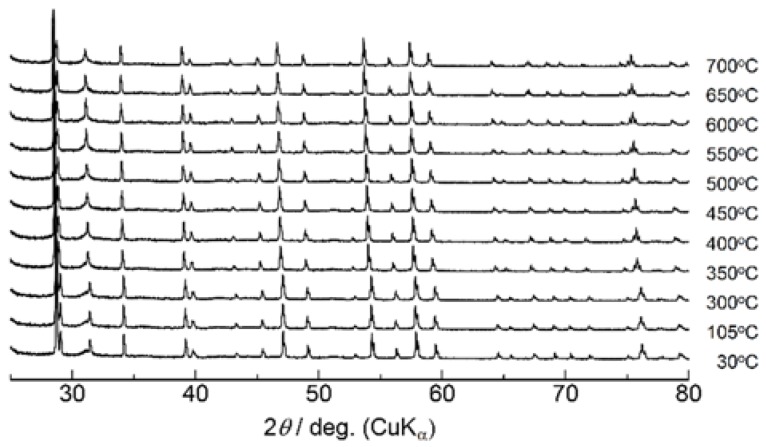
X-ray diffraction patterns of Ca_1−x_K_x_WO_4−x/2_ (*x* = 0.1) measured at high temperatures up to 700 °C.

**Figure 7:**
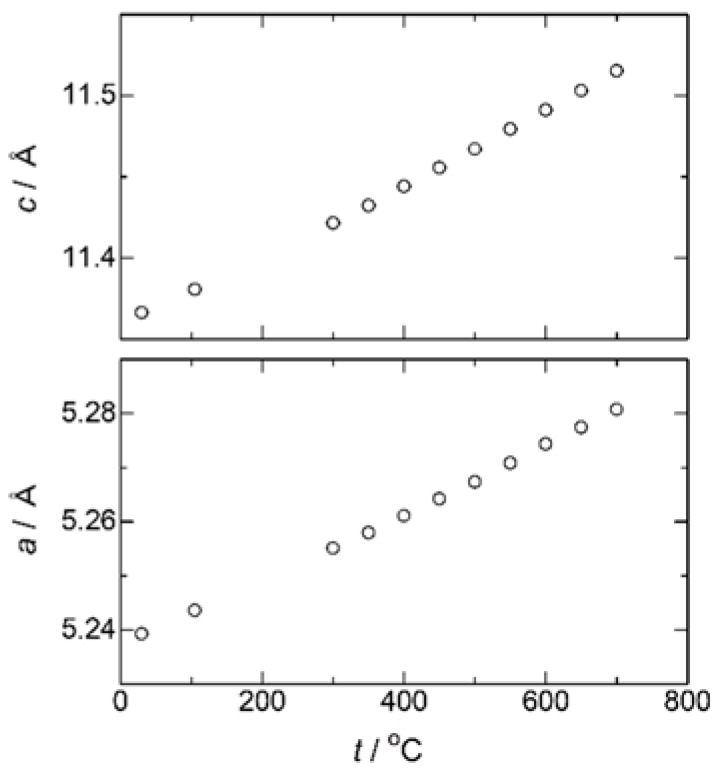
Temperature dependence of the lattice parameters of Ca_1−x_K_x_WO_4−x/2_ (*x* = 0.1).

**Figure 8:**
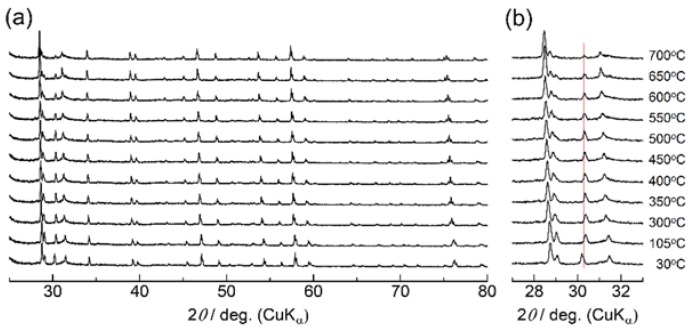
(**a**) High-temperature X-ray diffraction patterns of Ca_1−x_K_x_WO_4−x/2_ (*x* = 0.4); (**b**) Diffraction pattern enlarged in the range 27–33°.

High-temperature X-ray diffraction experiments were then performed focusing upon the conductivity jump accompanied by the endothermic heat at 500 °C. Figure 6 shows the XRD patterns of Ca_1−x_K_x_WO_4−x/2_ (*x* = 0.1) measured at various temperatures. While diffraction peaks shift toward a lower diffraction angle with temperature due to thermal expansion, any distinct change in peak was not observed around 500 °C. The lattice parameters obtained by the Rietveld refinements are plotted versus temperature in Figure 7. Both *a*- and *c*-length increase uniformly without any apparent discontinuity, suggesting that the conductivity jump observed around 500 °C is not accompanied by the drastic change in structure or apparent phase transition. On the other hand, for higher potassium introduction as *x* = 0.4, diffraction peaks of the second phase were detected at room temperature in Figure 8. Excess XRD peak at 2*θ* = 30.3° does not shift as other peaks associated with Scheelite-type structure, almost diminishing at 700 °C. This temperature is a little higher than that of the conductivity jump with the endothermic peak, which would be due to that undissolved potassium component, which remains even at the measurement of 700 °C. Assuming that the solution range becomes wider at higher temperatures, the residual second phase would dissolve into the Scheelite lattice above 500 °C to create oxide ion vacancies even for *x* = 0.4, which would directly contribute to the oxide ion conduction. Even though any excess diffraction peak was not detected for *x* = 0.2 at room temperature, dissolution of a very small amount of potassium ions into the Scheelite lattice might occur for the observed very small endothermic heat at 500 °C. In addition to the solid solution formation, subtle disordering of oxide ions should be considered, which would be made based on the neutron diffraction as a future work.

## 4. Conclusions

The solid solution range of Ca_1−x_K_x_WO_4−x/2_ is estimated as *x* ≤ 0.2 from the room-temperature X-ray diffraction experiments, while a slight amount of endothermic heat in DTA trace was observed around 500 °C on heating for *x* = 0.2. Above this temperature, oxide ion conduction appears to enhance the conductivity by more than three orders of magnitude. High-temperature oxide ion conductivity increases linearly with the potassium substitution beyond the room-temperature solid solution limit to achieve 0.01 Scm^−1^ at 600 °C for *x* = 0.4. High-temperature X-ray diffraction suggested any apparent structural change occurs for *x* = 0.1, while presumably K_2_WO_4_ of the second phase dissolves into the Scheelite lattice at 700 °C. K-substituted CaWO_4_ shows higher conductivity in comparison with the Cs-substituted system at the intermediate temperature of 600 °C.

## References

[1] Brian C.H., Angelika H. (2001). Materials for fuel-cell technologies. Nature.

[2] Goodenough J.B. (2003). Oxide-Ion Electrolytes. Annu. Rev. Mater. Res..

[3] Malavasi L., Fisher C.J., Islam M.S. (2010). Oxide-ion and proton conducting electrolyte materials for clean energy applications: Structural and mechanistic features. Chem. Soc. Rev..

[4] Galasso F.S. (1970). Structure and Properties of Inorganic Solids.

[5] Esaka T., Minaai T., Iwahara H. (1991). Oxide Ion Conduction in the Sintered Ceramics of the System Pb_1−x_La_x_WO_4+x/2_. Denki Kagaku.

[6] Esaka T., Mina-ai T., Iwahara H. (1992). Oxide ion conduction in the solid solution based on the scheelite-type oxide PbWO_4_. Solid State Ion..

[7] Takai S., Touda S., Oikawa K., Mori K., Torii S., Kamiyama T., Esaka T. (2002). Powder neutron diffraction study of Ln-substituted PbWO_4_ oxide ion conductors. Solid State Ion..

[8] Takai S., Satou M., Yoshida T., Chikashige N., Kita T., Esaka T. (2011). Conduction Property of PbWO_4_-and PbMoO_4_-based Oxide Ion Conductors in Lower Oxygen Partial Pressures. Electrochemistry.

[9] Takai S., Morhishita Y., Kondo Y., Yao T., Yabutsuka T., Esaka T. (2016). Electrochemical properties of Cs-substituted CaWO_4_ and BaWO_4_ oxide ion conductors. J. Ceram. Soc. Jpn..

[10] Shannon R.D. (1976). Revised effective ionic radii and systematic studies of interatomic distances in halides and chalcogenides. Acta Cryst. A.

[11] Izumi F., Momma K. (2007). Three-dimensional visualization in powder diffraction. Solid State Phenom..

